# Fishery-Induced Selection for Slow Somatic Growth in European Eel

**DOI:** 10.1371/journal.pone.0037622

**Published:** 2012-05-22

**Authors:** Daniele Bevacqua, Fabrizio Capoccioni, Paco Melià, Simone Vincenzi, José M. Pujolar, Giulio A. De Leo, Eleonora Ciccotti

**Affiliations:** 1 Dipartimento di Scienze Ambientali, Università degli Studi di Parma, Parma, Italy; 2 INRA, UR1115 PSH, Avignon, France; 3 Dipartimento di Biologia, Università Roma Tor Vergata, Roma, Italy; 4 Dipartimento di Elettronica e Informazione, Politecnico di Milano, Milano, Italy; 5 Centre for Stock Assessment Research, University of California Santa Cruz, Santa Cruz, California, United States of America; 6 Dipartimento di Biologia, Università degli Studi di Padova, Padova, Italy; 7 Hopkins Marine Station, Stanford University, Stanford, California, United States of America; Institute of Marine Research, Norway

## Abstract

Both theoretical and experimental studies have shown that fishing mortality can induce adaptive responses in body growth rates of fishes in the opposite direction of natural selection. We compared body growth rates in European eel (*Anguilla anguilla*) from three Mediterranean stocks subject to different fishing pressure. Results are consistent with the hypotheses that *i*) fast-growing individuals are more likely to survive until sexual maturity than slow-growing ones under natural conditions (no fishing) and *ii*) fishing can select for slow-growing individuals by removing fast-growing ones. Although the possibility of human-induced evolution seems remote for a panmictic species like such as the European eel, further research is desirable to assess the implications of the intensive exploitation on this critically endangered fish.

## Introduction

Fast body growth is traditionally interpreted as a beneficial trait in fish, according to the belief that “faster is better” (e.g. [1]). In general, natural mortality rates are negatively linked to body size via allometric relationships (e.g. [2], [3]), and rapid body growth shortens the duration of the permanence of fish in the most vulnerable size classes before reproduction. However, the “faster is better” hypothesis has been challenged by recent studies showing that body growth itself has a physiological trade-off with other vital rates [4].

Fishing mortality can largely exceed natural mortality and has a strong size-selective effect, as most fisheries preferentially target larger and/or faster growing individuals [5]. Fishing can induce adaptive responses, intended to increase fitness, in body growth rates, resulting from changes in gene frequency across generations (adaptive evolution) or from changes in phenotypic distribution over time, without genetic change (adaptive plasticity). In particular, a decline in average body growth rate has been predicted as a likely response to selective removal of fast-growing individuals by fisheries [5].

In the last decades, the dramatic decline of the global European eel (*Anguilla anguilla*) stock has raised worldwide concern. Phenotypic plasticity in body growth rate is extremely high in European eel [6], [7]. However, no study so far has explored the possible existence of different selective pressures on body growth in different environments. In the present study, we compare body growth rates in eels from three Mediterranean stocks subject to different exploitation levels to test *i*) if fast-growing individuals have higher survival during the pre-reproductive growing phase in natural conditions and *ii*) if fishing pressure can select for slower body growth of spawners.

## Materials and Methods

The European eel is a semelparous, catadromous and panmictic species exploited across its entire distribution area at different life stages [8]. After spawning in the Sargasso Sea, eel larvae are carried by oceanic currents towards the continental shelf of Europe and North Africa, metamorphose into glass eels and settle in continental waters, where they feed and grow during the so-called pre-reproductive yellow phase. Eels are sexually undifferentiated at the beginning of the yellow phase and germ cells start differentiating in individuals >20 cm [9]. When eels reach the maturation size (ca. 45 cm for males and 60 cm for females), they undergo a metamorphosis to the silver stage, stop eating and growing and start their migration back to the Sargasso Sea, where they mate, spawn and eventually die [10]. As maturation rate is size rather than age-dependent [11] and body growth is affected by high inter-individual variability, silver eels escaping each year from continental waters are not homogeneous with respect to age.

**Figure 1:**
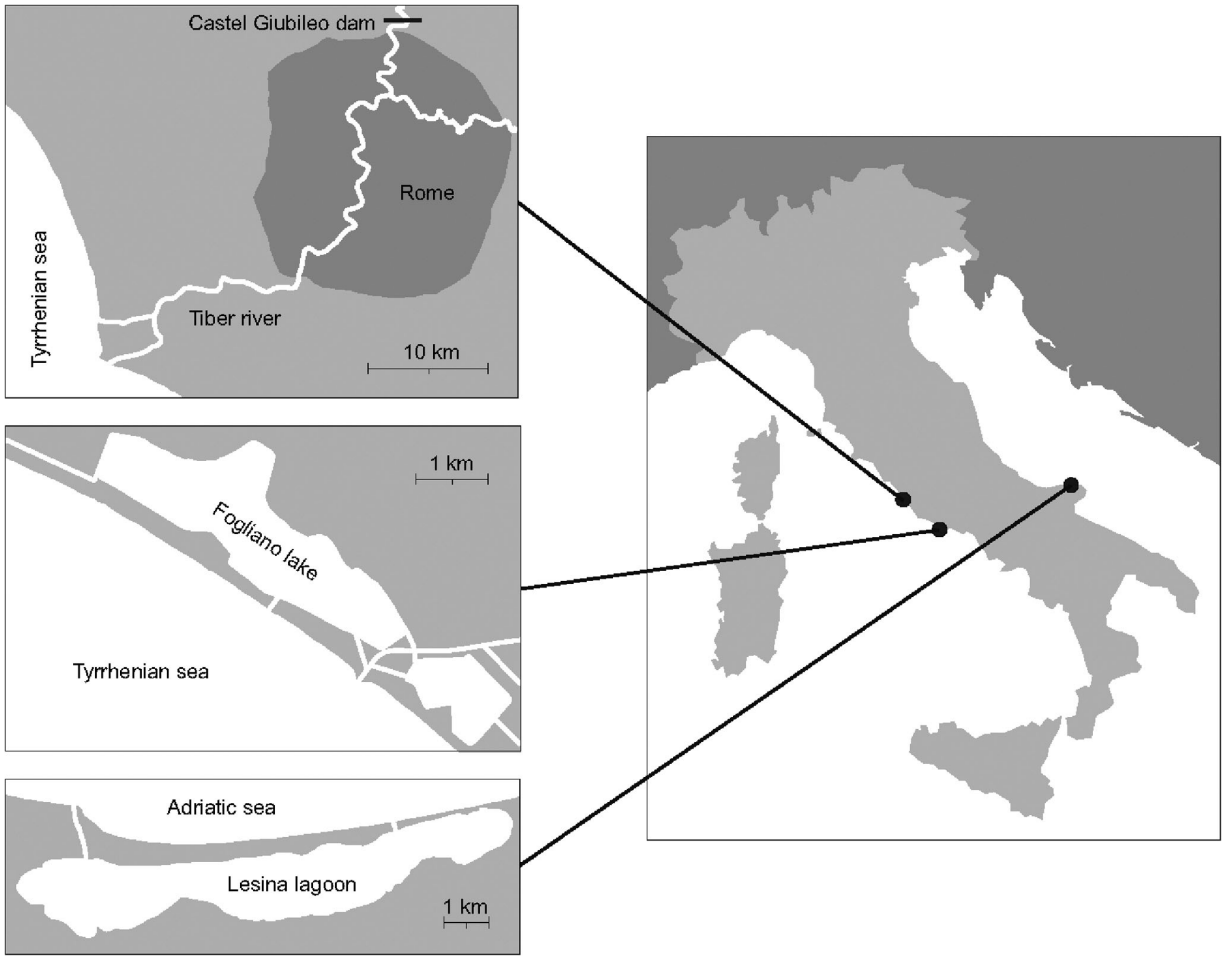
Study sites. Location of the three study sites (lower Tiber river, Fogliano lake and Lesina lagoon).

Between 2007 and 2009, we sampled yellow and silver eels at three distinct Mediterranean sites (Fig. 1) characterized by different levels of fishing pressure: *i*) the low course of the Tiber river (TIB), *ii*) the Fogliano lake (FOG), and *iii*) the Lesina lagoon (LES). In TIB, the fishery mainly targets small yellow eels (<40 cm) that are then sent to aquaculture facilities [12]. In FOG, eel exploitation is prohibited. In LES, fyke nets (which have a larger mesh size than those used in TIB) intercept silver eels >40 cm [13]. Eels were collected by means of both commercial and experimental fyke nets. This allowed us to obtain also individuals smaller than those commonly caught by commercial fisheries. We sacrificed, measured for total length (*L*) and aged through otolith reading a total of 1210 eels (*N*_TIB_ = 471; *N*_FOG_ = 273; *N*_LES_ = 466). We determined sex and maturation stage by histological examination of gonads [14] and computed Pankhurst ocular index (OI; cf. [15]). To test for possible selection for fast/slow body growth during the early yellow phase, we compared the mean body growth rate between age 1 and 3 of sub-adult and silver eels. The “sub-adult” group included individuals with *L*≤35 cm, OI≤6.5 and gonads with undifferentiated germ cells. The “silver” group included individuals with OI>6.5 and fully differentiated testis or ovaries. All the silver individuals were above the cut-off length of 35 cm. As the potential for body growth (i.e. being a slow or a fast grower) is maintained through the whole yellow phase [6], we assumed that the growth rate distribution of sub-adults reflects the original variability of this vital trait within a stock (before selection), while the growth rate distribution of silver eels reflects the residual variation (after selection), namely the one that better fits under the set of selective (natural and/or anthropogenic) forces playing at a specific site.

To test these hypotheses, we back-calculated body length *L_i_* at age *i* for individuals belonging to the “sub-adult” and “silver” groups according to the “biological intercept” method [16]:
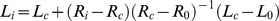
(1)where *L_c_* is body length at capture, *R_i_*, *R_c_* and *R*_0_ are the otolith radii at annulus *i*, at capture time and at the beginning of the continental phase (which corresponds to an initial body length *L*_0_ = 7 cm).

Finally, we computed body growth rate g between age 1 and 3 for each individual as:
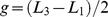
(2)(see Online Supporting Information, Text S1 for details).

For each stock (TIB/FOG/LES) and group (sub-adult/silver), we assessed the uncertainty in the estimate of mean growth rates via bootstrapping, with data resampled 10,000 times, and computed confidence intervals with the percentile method. As for silver individuals, we analyzed males and females separately to account for possible differences in body growth caused by sexual dimorphism, because silver males are systematically smaller than silver females. For each of the three stocks, we tested for differences in body growth rate between sub-adult and silver individuals through the following randomization test:

1. we calculated the absolute difference between the mean growth rate of sub-adults, gsa, and that of silver individuals, gs:

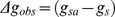
(3)where subscript “obs” indicates that the value has been computed on observed data. The null hypothesis is that 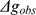
does not significantly differ from what would be expected by chance (i.e. if there were no difference between the early growth rate experienced by sub-adult and silver individuals) and the alternative hypothesis is that it does;

1. we randomly re-assigned the “sub-adult” and “silver” label to each individual and calculated the sample statistic 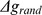
for this randomized dataset. The subscript “rand” indicates that, in this case, the value has been calculated from the randomized data. We replicated this reshuffling procedure 10,000 times, obtained a vector of 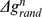
(n = 1, 2, …10,000), and derived an empirical null distribution for 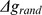;
2. from this empirical distribution, we computed the p-value for the null hypothesis (i.e. the probability that the observed difference in growth rates between groups is due to chance) as the proportion of 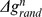
that are greater than or equal to 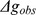.

To increase the accuracy of the randomization test, we carried out statistical analyses only on groups including at least 10 individuals. Since sub-adult and silver individuals belong to different cohorts, we run a one-way ANOVA (see OSI; Table S1 and Figure S1) to test for possible inter-cohort variation of body growth caused by environmental factors not explicitly accounted for in the analysis. Finally, we tested through a regression analysis (see OSI; Table S2 and Figure S2) whether growth rate affects maturation size.

## Results

The sub-adult group included 103, 33 and 22 sexually undifferentiated individuals in TIB, FOG and LES, respectively. Silver males were 86, 48 and 5, while silver females were 8, 47 and 42 in TIB, FOG and LES, respectively. Only 33% of the 1210 sacrificed eels could be classified as sub-adults or silver, as most individuals were sexually differentiated but still in the yellow stage.

**Figure 2:**
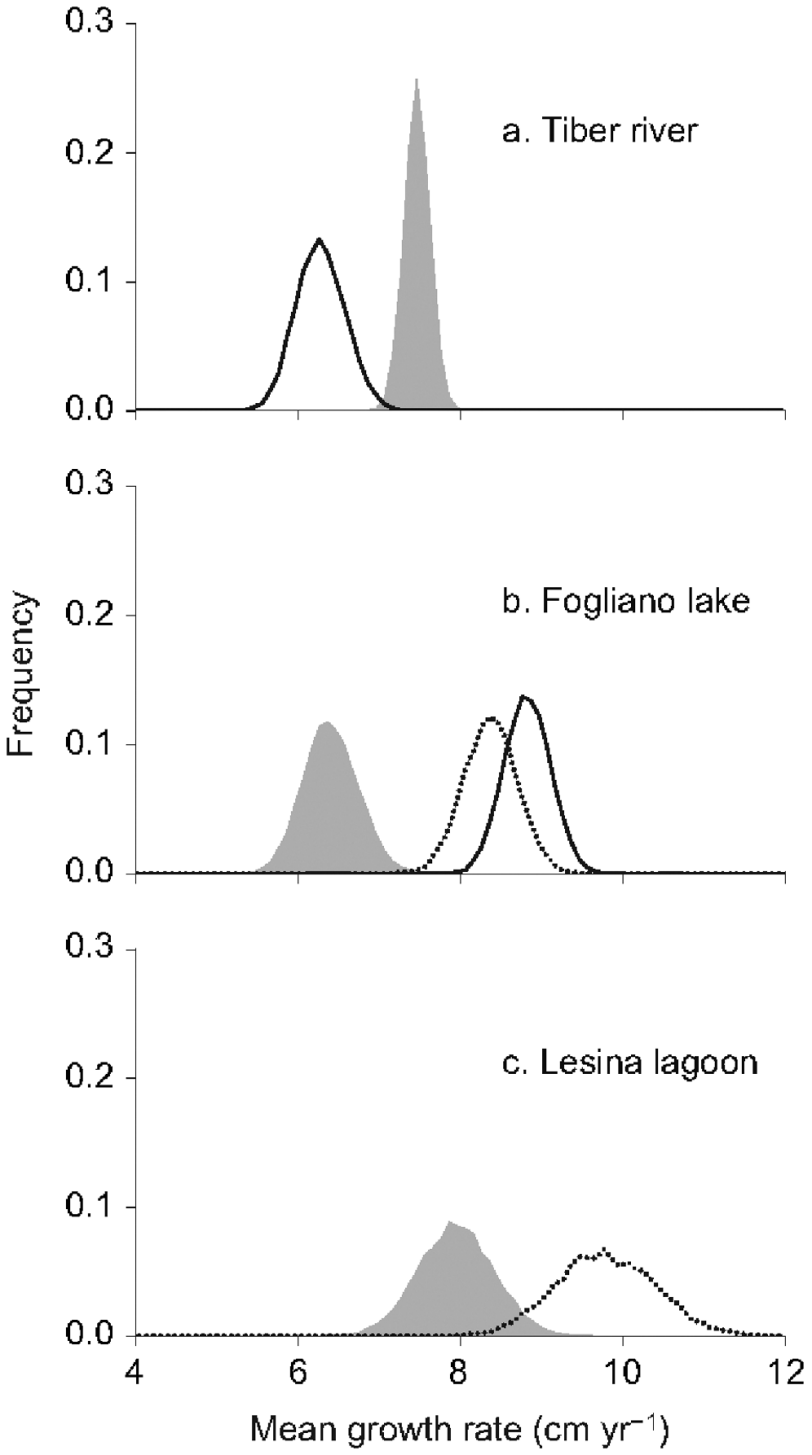
Growth rate distribution of sub-adults, silver males and silver females. Empirical bootstrap distribution of body growth rate (population mean) between age 1 and 3 of sub-adult European eels (shaded areas), silver males (solid lines) and silver females (dotted lines) sampled at three Mediterranean sites: a Tiber river, b Fogliano lake and c Lesina lagoon.

Individual body growth rates between age 1 and 3 (eq. 2) varied between 1.8–17.5 (TIB), 3.5–12.7 (FOG), and 3.9–19.5 (LES) cm yr^−1^. The bootstrapped distribution of mean growth rate (population mean) are reported in Fig. 2 for the three stocks. Average growth rate of sub-adults from TIB, FOG and LES was equal to 7.5 (90% CI 7.2–7.7), 6.4 (5.9–7.0) and 7.9 (7.2–8.7) cm yr^−1^, respectively, while for silver individuals it was 6.3 (5.8–6.8) cm yr^−1^ for silver males in TIB, 8.8 (8.3–9.3) and 8.4 (7.8–8.9) cm yr^−1^ for silver males and females in FOG, respectively, and 9.8 (8.8–10.8) cm yr^−1^ for silver females in LES.

The randomization test showed that mean growth rate in TIB was significantly lower in silver (male) eels than in sub-adults (*P*<0.01). In contrast, in FOG the growth rates of both male and female silver eels were significantly higher than that of sub-adults (*P*<0.01), while in LES (where only silver eels are fished) the difference between silver (female) eels and the sub-adult sample were barely above statistical significance (*P* = 0.06). Body growth rate did not vary among cohorts in TIB (*P* = 0.20) and FOG (*P* = 0.35), while it varied in LES (*P*<0.01; see OSI for details). There was no significant between eel body growth rate and body size at silvering, irrespective of sex and site (see OSI).

## Discussion

Results confirm the high plasticity of eel body growth, with fast-growing individuals that can grow up to 10 times more rapidly than slow-growing ones (e.g. 17.5 vs. 1.8 cm yr^−1^ in TIB). At FOG, where the local eel stock is unexploited, fast-growing eels have a higher probability to survive the pre-reproductive stage. In most fish species, this is generally due to the fact that fast-growing individuals attain larger sizes more rapidly and are therefore less susceptible to predation [1]. In our case, predation is likely to play a minor role as a driver of selection, because eels are generally top predators (but see [17]). Instead, the pattern we observed may be better explained in terms of higher competitive ability of fast growers. Superiority in interference competition is known to be largely determined by body size [18]. In eel species, fast growth is known to be favorable in aquaculture conditions, where fast-growing individuals can reduce feeding rates of slow-growing ones and often show cannibalistic behavior [10]. Our results suggest that the “faster is better” hypothesis may hold for *A. anguilla* also in the wild, provided that fishing pressure is absent.

On the other hand, when yellow eels are subject to high fishing pressure, as in the case of TIB, the silver group is composed by slower-growing individuals. Higher vulnerability to fishing of fast-growing eels might be related to their behavior. In a whole-lake manipulative experiment conducted by Biro & Post [19] on rainbow trout *Oncorhynchus mykiss*, fast-growing individuals showed more aggressive feeding behavior, which increased their capture rates. An analogous mechanism (more aggressive behavior leading to fast growth and higher catchability) might explain the patterns observed in our study. Different growth rates in eels can reflect feeding preferences: fast-growing individuals are mostly piscivorous and must actively chase their prey, while slow-growing ones usually feed on benthic organisms, whose capture requires a less active behavior [10]. Therefore, also in the case of eels, a trait providing a competitive advantage in unexploited conditions may become a shortcoming under heavy fishing pressure, as envisioned by Biro & Post [19].

Results from LES suggest that a fishery targeting silver eels does not override natural selection towards fast growers. This result is not surprising, as harvested silver eels have already concluded their growing phase. In this respect, the LES study site is more similar to the unexploited FOG site, in terms of selective pressure exerted on growth rate.

Inter-annual environmental variation (e.g. food availability and distribution, temperature, salinity) may be responsible for observed changes in growth rates [20]. However, growth rates computed for different cohorts of yellow eels in TIB and FOG showed no significant inter-cohort differences, a result that partially rules out the possibility that environmental factors may have caused short-term changes in body growth. Of course, it remains the possibility that older cohorts of silver eels might have experienced substantially different conditions in their early life phase with respect to the more recently recruited sub-adults. If this were the case, differences in growth rate distributions would not be due only to fishing pressure, but also to other environmental factors we could not control for. Unfortunately, there are no samples dating back to the early period of permanence in inland waters of the oldest silver eels. As a consequence, there is no way to formally test whether older eels experienced different environmental conditions in their early years of life with respect to what experienced by younger cohorts of yellow eels in the late years of the last decade. However, there is no evidence that environmental conditions have dramatically changed in any of the three sites during the last twenty years [21]–[24]. The only exception is for water salinity in LES, which underwent wide fluctuations in the last decade (between 15 and 25 [25], [26]). The significant inter-cohort variation observed in the growth rate of sub-adults from LES might reflect the influence of water salinity on body growth [27]. In this specific case, our analysis could have been biased by the changed conditions and we cannot reach a definitive conclusion with respect to this site.

A better insight into the selective force of yellow eel fishery on growth rate will be achieved only by future long-term experiments gathering information not just on eel growth, age and size at maturity, but also on historical series of environmental variables. A text-book reference case with respect to this issue is the study conducted by Swain et al. [28], who included proxies for inter-annual changes of the biotic and abiotic environment (i.e. stock density and temperature) and estimated their effect on growth rates, together with the effect of fishery selection on a 30-year historical series.

In conclusion, despite the abovementioned limitations of our study, results are consistent with the hypothesis that intensive fishing during the growing phase preferentially removes fast-growing eels. Studies on other exploited fish stocks reported that evolutionary changes in response to selective exploitation can result in reduced population productivity even when the stock is released from fishing pressure, hence reducing the capacity for population recovery [29]. In eel stocks released from fishing pressure, fishery-induced selection towards slower growth would determine less productivity of spawners, as individuals would be more susceptible to competition, while per capita fertility would not be affected, because maturation size is not linked to growth rate (as suggested by our results: see OSI). The possibility of a fishery-induced evolution of body growth seems remote for a panmictic species such as the European eel, at least as long as a sufficient amount of spawners come from unexploited stocks. However, considering the critical state of this species, the open debate on the processes leading to its current decline, and according to the precautionary principle [30] our findings urge further research to investigate the impacts that the widespread and systematic selection for smaller, slow-growing eels might have on the reproductive output of the spawning stock.

## Supporting Information

Table S1 Results of one-way ANOVA of body growth rate with respect to cohort for the three study sites (TIB: Tiber river; FOG: Fogliano lake; LES: Lesina lagoon).
(DOC)

Table S2 Regression analysis of body length of silver eels *vs*. body growth rate for the three study sites (TIB: Tiber river; FOG: Fogliano lake; LES: Lesina lagoon).
(DOC)

Figure S1 Box-whisker plots of individual body growth rate for different cohorts at a) Tiber river, b) Fogliano lake and c) Lesina lagoon. Lines within the boxes represent lower quartile, median and upper quartile values. Vertical dashed lines extend from quartile values to the most extreme data value within 1.5 times interquartile range. Outliers are plotted individually.
(DOC)

Figure S2 Total length (cm) of silver eels against body growth rate (cm/yr) and relevant regression lines for a) Tiber river, b) Fogliano lake and c) Lesina lagoons. Triangles and circles indicate males and females, respectively.
(DOC)

Text S1 (DOC)
