# Supplementary material for: Fishery-Induced Selection for Slow Somatic Growth in European Eel
Source: PLoS One. 2012 May 22;7(5):e37622. doi: 10.1371/journal.pone.0037622 (PMC3358250; doi:10.1371/journal.pone.0037622)
Supplement: Table S1 — Results of one-way ANOVA of body growth rate with respect to cohort for the three study sites (TIB: Tiber river; FOG: Fogliano lake; LES: Lesina lagoon). (DOC) [file pone.0037622.s001.doc]

**Table S1. Results of one-way ANOVA of body growth rate with respect to cohort for the three study sites (TIB: Tiber river; FOG: Fogliano lake; LES: Lesina lagoon).**

| **Site** |  | **d. f.** | **Sum. sq.** | **Mean sq.** | ***F* ratio** | ***P*** |
| --- | --- | --- | --- | --- | --- | --- |
| TIB | cohort | 1 | 3.51 | 3.51 | 1.65 | 0.2 |
|  | residuals | 94 | 199.7 | 2.13 |  |  |
| FOG | cohort | 1 | 3.52 | 3.51 | 0.92 | 0.35 |
|  | residuals | 17 | 64.8 | 3.81 |  |  |
| LES | cohort | 1 | 28.74 | 28.74 | 13.02 | < 0.01 |
|  | residuals | 17 | 37.52 | 2.21 |  |  |
