# Supplementary material for: Fishery-Induced Selection for Slow Somatic Growth in European Eel
Source: PLoS One. 2012 May 22;7(5):e37622. doi: 10.1371/journal.pone.0037622 (PMC3358250; doi:10.1371/journal.pone.0037622)
Supplement: Table S2 — Regression analysis of body length of silver eels vs. body growth rate for the three study sites (TIB: Tiber river; FOG: Fogliano lake; LES: Lesina lagoon). (DOC) [file pone.0037622.s002.doc]

**Table S2. Regression analysis of body length of silver eels *vs*. body growth rate for the three study sites (TIB: Tiber river; FOG: Fogliano lake; LES: Lesina lagoon).**

| **Site** |  | **d. f.** | **Sum. sq.** | **Mean sq.** | ***F* ratio** | ***P*** |
| --- | --- | --- | --- | --- | --- | --- |
| TIB, males | growth rate | 1 | 3.52 | 3.52 | 0.46 | 0.5 |
|  | residuals | 84 | 647.51 | 7.71 |  |  |
| FOG, males | growth rate | 1 | 0.42 | 0.42 | 0.18 | 0.67 |
|  | residuals | 46 | 106.79 | 2.31 |  |  |
| FOG, females | growth rate | 1 | 221.5 | 221.46 | 2.67 | 0.11 |
|  | residuals | 45 | 3730.9 | 82.91 |  |  |
| LES, females | growth rate | 1 | 86.76 | 86.76 | 1.39 | 0.25 |
|  | residuals | 40 | 2506.6 | 62.67 |  |  |
