# Supplementary material for: Fishery-Induced Selection for Slow Somatic Growth in European Eel
Source: PLoS One. 2012 May 22;7(5):e37622. doi: 10.1371/journal.pone.0037622 (PMC3358250; doi:10.1371/journal.pone.0037622)
Supplement: Figure S1 — Box-whisker plots of individual body growth rate for different cohorts at a) Tiber river, b) Fogliano lake and c) Lesina lagoon. Lines within the boxes represent lower quartile, median and upper quartile values. Vertical dashed lines extend from quartile values to the most extreme data value within 1.5 times interquartile range. Outliers are plotted individually. (DOC) [file pone.0037622.s003.doc]

1. Online Supporting Information (OSI)

## Supplementary figure

**Figure S1**. Box-whisker plots of individual body growth rate for different cohorts at a) Tiber river, b) Fogliano lake and c) Lesina lagoon. Lines within the boxes represent lower quartile, median and upper quartile values. Vertical dashed lines extend from quartile values to the most extreme data value within 1.5 times interquartile range. Outliers are plotted individually.
