# Supplementary material for: Fishery-Induced Selection for Slow Somatic Growth in European Eel
Source: PLoS One. 2012 May 22;7(5):e37622. doi: 10.1371/journal.pone.0037622 (PMC3358250; doi:10.1371/journal.pone.0037622)
Supplement: Figure S2 — Total length (cm) of silver eels against body growth rate (cm/yr) and relevant regression lines for a) Tiber river, b) Fogliano lake and c) Lesina lagoons. Triangles and circles indicate males and females, respectively. (DOC) [file pone.0037622.s004.doc]

1. Online Supporting Information (OSI)

## Supplementary figure

**Figure S2**. Total length (cm) of silver eels against body growth rate (cm/yr) and relevant regression lines for a) Tiber river, b) Fogliano lake and c) Lesina lagoons. Triangles and circles indicate males and females, respectively.
