# Supplementary material for: Fishery-Induced Selection for Slow Somatic Growth in European Eel
Source: PLoS One. 2012 May 22;7(5):e37622. doi: 10.1371/journal.pone.0037622 (PMC3358250; doi:10.1371/journal.pone.0037622)
Supplement: Text S1 — (DOC) [file pone.0037622.s005.doc]

1. Online Supporting Information (OSI)
2. Fishery-induced Selection for Slow Somatic Growth in European Eel

D. Bevacqua, F. Capoccioni, P. Melià, S. Vincenzi, J.M. Pujolar, G.A. De Leo and E. Ciccotti

# Supplementary materials and methods

## Otolith reading and back-calculation of individual body growth

Otoliths were extracted, embedded in epoxyn resin and stained with 3% toluidine blue after grinding the convex side. We determined fish age by reading annual otolith rings (annuli) through a light stereomicroscope, from the first growth check (age 1^+^) outside the so-called zero band. This band is commonly assumed as the beginning of eel's continental growth [1,2]

Since body growth during the first year of continental life is difficult to determine, due to the absence of a clear relationship between otolith radius and body length [3], we computed the early growth rate of each individual as the average annual increment in body length experienced between age 1 and 3.

## Testing for inter-annual differences in body growth

As the comparison of body growth rates between sub-adult and maturing eels is based on data from individuals belonging to different cohorts, the results might in principle be affected by possible variation of environmental or demographic conditions experienced at recruitment and/or during the early growth phase. Therefore, we tested for possible differences in early body growth from cohort to cohort.

To avoid possible biases in the comparison caused by the migration of faster-growing individuals (which can determine the underestimation of body growth rates in older age classes) and by the size-selectivity of fishing, we restricted the analysis to individuals between 3 and 4 years of age, and measuring <35 cm (total length). For each individual we computed body growth rate *g* (eqn. 2) and year of recruitment *t_r_* = *t_c_* – *a_c_* (where *t_c_* is the year of capture and *a_c_* is the age at capture). Then, we performed a one-way analysis of variance (ANOVA) of body growth rate with cohort as a factor (for each stock separately). Results are summarized in Table S1 and Figure S1.

## *Testing for the effect of body growth rate on maturation size*

In order to test whether there is a significant linear relationship between body growth and body size at maturation, we performed a regression analysis between the body length of silver eels and their back-calculated body growth rate. To account for sexual dimorphism and possible variability from site to site, we performed separate analyses for males and females and for the three stocks. Results are summarized in Table S2 and Figure S2.

## Supplementary references

1. Poole WR, Reynolds JD, Moriarty C (2004) Early post-larval growth and otolith patterns in the eel *Anguilla anguilla*. Fish Res 66: 107–114.
2. ICES (2009) Annex 4: manual for the ageing of Atlantic eel. In ICES Workshop on Age Reading of European and American Eel (WKAREA), 20–24 April 2009, Bordeaux, France. ICES CM 2009:ACOM 48.
3. Francis RICC (1990) Back-calculation of fish length: a critical review. J Fish Biol 36: 883–902.

**Ethic Statement**

***Eel sampling was carried out within the biological monitoring required by the Italian national program for the collection of fishery data 2009-2010 (developed according to EC Regulation n. 199/2008) and authorized by the Italian Ministry of Agricultural, Food and Forestry Policies, General Directorate of Fisheries and Aquaculture with Ministerial Decree n.34/2009. Sampling in Fogliano lake was further authorized by Ente Parco Nazionale del Circeo (authority of the local national park) and Ufficio Territoriale per la Biodiversità di Fogliano (under the authority of the State Forestry Corps) with a specific authorization (PNC/DIR/2009/1586 – 26/05/2009). In presence of these authorizations, the authorization of the Ethics Committee of University of Rome Tor Vergata was not required. The authors declare that all relevant ethical safeguards have been met in relation to animal killing, as each fish was anaesthetized with 2-phenoxyethanol 1% and then painlessly sacrificed.***
